# Long-term results of Sauvé–Kapandji procedure

**DOI:** 10.1177/17531934211004459

**Published:** 2021-04-12

**Authors:** Lisa Reissner, Andreas Schweizer, Ines Unterfrauner, Lea Estermann, Ladislav Nagy

**Affiliations:** Department of Orthopedics, Hand Surgery Division, Balgrist University Hospital Zurich, Switzerland

**Keywords:** Distal radioulnar joint, Sauvé–Kapandji, complication, ulnar stump instability, radioulnar convergence, sonography

## Abstract

The Sauvé–Kapandji procedure is an established treatment option for distal radioulnar joint dysfunction. We retrospectively analysed 36 patients following Sauvé–Kapandji procedure between 1997 and 2013. Fifteen patients were available for a follow-up after a mean of 13 years (range 6 to 23). Six patients needed revision surgery because of ulnar stump instability. Radiographs and sonography were performed to quantify the instability of the proximal ulnar stump. These showed a radioulnar convergence of 8 mm without weight and 2 mm while lifting 1 kg. Sonographically, the proximal ulnar stump dislocated by 8 mm to the volar side while applying pressure to the palm, compared with 4 mm on the contralateral side. Sonographically measured ulnar stump instability showed a positive strong correlation with the Disabilities of the Arm, Shoulder and Hand questionnaire and Patient-Reported Wrist Evaluations and a negative strong correlation with grip strength and supination torque. Because of the high incidence of revision surgery due to instability of the proximal ulnar stump, we restrict the use of the Sauvé–Kapandji procedure only to very selected cases.

**Level of evidence:** IV

## Introduction

Several surgical procedures have been proposed for the management of distal radioulnar joint (DRUJ) osteoarthritis, such as the Darrach procedure (Darrach, 1913), the Sauvé-Kapandji (SK) procedure (Sauvé and Kapandji, 1936), hemiresection interposition arthroplasty (Bowers, 1985), matched distal ulna resection (Watson et al., 1986) and implant arthroplasty (Herbert and van Schoonhoven, 2007; Masaoka et al., 2002; Reissner et al., 2016; Scheker et al., 2001).

The SK procedure combines a DRUJ arthrodesis with the creation of a distal ulnar pseudarthrosis for the salvage of DRUJ dysfunction (Sauvé and Kapandji, 1936). Instability of the proximal ulnar stump and radioulnar convergence resulting in a painful ulnar stump have been noted (Minami et al., 1995; Nakamura et al., 1992). The incidence and the impact of the ulnar stump instability remains unknown, as this complication is not reported consistently. Nevertheless, numerous soft tissue techniques, including the use of the flexor carpi ulnaris (FCU) (del Pino and Fernandez, 1998; Lamey and Fernandez, 1998), extensor carpi ulnaris (ECU) (Chu et al., 2008; Minami et al., 2000, 2006), a combination of both (Breen and Jupiter, 1989) or allograft (Sotereanos et al., 2014) have been described to stabilize the unstable proximal ulnar stump. We report our results after a mean 13-year (range 6 to 23) follow-up of patients who underwent the SK procedure with specific focus on the instability of the ulnar stump.

## Methods

Thirty-six patients with SK procedures between 1997 and 2013 at a tertiary university hospital were screened out. Fifteen patients (16 wrists, 11 men and four women with a mean age of 59 years; range 18 to 70) were available for a follow-up consultation. Twenty-one patients had to be excluded because they lived abroad (*n* = 8), had died (*n* = 7), declined to participate (*n* = 3) or were unavailable (*n* = 3). The most common indications for the SK procedure were post-traumatic arthritis (*n* = 12), rheumatoid arthritis (*n* = 1) and primary osteoarthritis (*n* = 2). Baseline and demographic data, including diagnosis as well as date and type of surgeries, were retrospectively extracted from patient records (Table S1). All patients with a time interval to surgery of at least 6 years were invited to participate in a scheduled clinical and radiographic follow-up examination. The study was approved by the local ethics committee, and all patients provided written informed consent for their data to be used for this analysis.

### Surgical technique

All SK procedures were performed by four senior hand surgeons. One surgeon has more than 30 years of experience in hand surgery, has published extensively about the DRUJ and is a member of the International Wrist Investigator Workshop. The expertise level of this surgeon was Grade 4; two other surgeons have more than 20 years of experience in hand surgery (Level 3) and the fourth surgeon with hand surgery experience of more than 10 years was Grade 2, according to the criteria of Tang (Tang, 2009; Tang and Giddins, 2016).

A dorsal incision was made over the distal ulna, the fifth extensor compartment was opened, the extensor digit minimi was retracted, and the capsule incised. The DRUJ was decorticated, and an osteotomy of the ulna was performed proximal to the metaphysis to allow for a 1 cm pseudarthrosis. One or two cannulated 3.5 mm screws were placed across the DRUJ. Bone graft from the resected ulna was packed into the arthrodesis site. The ulnar stump was stabilized using a retinaculum flap (*n* = 4) or a partial FCU tenodesis (*n* = 8). The remaining four wrists were not additionally stabilized. Stabilization of the ulna stump with the FCU was performed by harvesting a distally based strip of the FCU (50%) and weaving it through a drill hole in the proximal ulna. Stabilization with a retinaculum flap was done by raising a strip of extensor retinaculum based at its ulnar border next to the pisiform. The free end was inserted into the proximal ulnar stump in the same manner as the FCU. Finally, the extensor digit minimi tendon was repositioned, and the extensor retinaculum and skin were sutured. All patients wore a removable splint above the elbow for 3 weeks and below-the-elbow splint for another 3 weeks and then began active movements 6 weeks after surgery.

### Subjective and functional assessment

Subjective assessment was based on a visual analogue scale (VAS) for pain (on a scale of 0 to 10 for severity), the Disabilities of the Arm, Shoulder and Hand (DASH) questionnaire (Hudak et al., 1996) and the Patient-Rated Wrist Evaluation (PRWE) score (MacDermid et al., 1998). Thereby, clinical scores were compared between patients with revision surgery after SK and those without.

**Figure 1.**
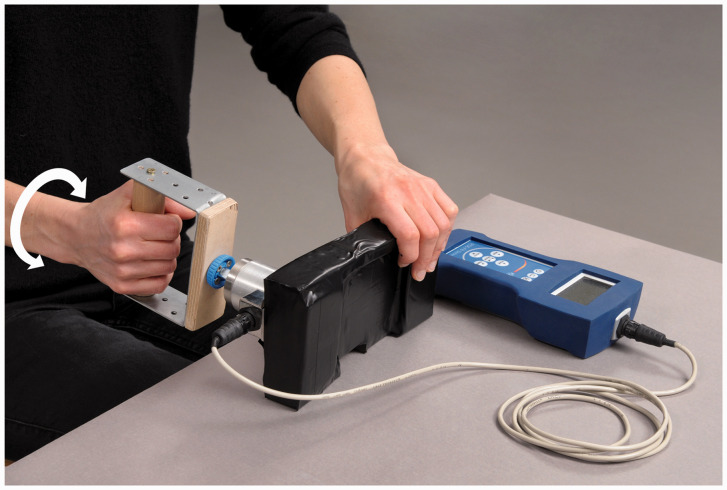
Torque-force measurement devise.

The range of motion (ROM) of the wrist was determined using a goniometer. Results were compared between the pre- and postoperative conditions. Grip strength was measured with a Jamar dynamometer and compared with the contralateral hand. In addition, the force during pronation and supination was measured with a torque force device and compared with the contralateral side (Figure 1). The ability to return to work after the operation was recorded.

### Radiographic parameters

Posteroanterior and lateral radiographs of the affected wrist were taken to assess DRUJ nonunion and presence or absence of scalloping at the radius on the posteroanterior view. The interosseous distance between the radius and ulna at the level of the distal end of the proximal ulnar stump was measured on the posteroanterior films as described by Nakamura et al. (1992). A second radiograph was made with the forearm suspended in the air and the elbow flexed 90° while lifting 1 kg and 2 kg weights to assess the radioulnar convergence in order to quantify the instability (Lees and Scheker, 1997; Scheker, 2008).

### Ultrasound examination

**Figure 2.**
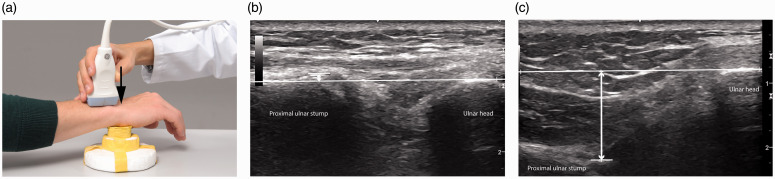
Sonography to assess the stability of the DRUJ of the operated hand (a). Measurement of the distance between the dorsal surface of the proximal ulnar stump and the surface of the ulnar head longitudinally (b) and while actively pressing (c) the volar surface of the hand onto the block.

Ultrasound was performed to quantify the ulnar stump instability and compare it with the contralateral side. All ultrasound assessments were performed by a hand surgeon who has a certificate of sonography of the hand and has more than 6 years’ experience in musculoskeletal ultrasound. The expertise level of the examiner was Grade 3 according to the criteria of Tang (Tang, 2009; Tang and Giddins, 2016). All ultrasound examinations were performed with a high-resolution linear array transducer with 12 MHz  frequency range (LOGIQ e R6, GE Medical Systems, Wuxi, China). During sonography, the patients were assessed with particular attention to the instability of the proximal ulnar stump (Hess et al., 2012). We measured the distance between the dorsal surface of the ulnar stump and the surface of the ulnar head longitudinally. Measurements were done while actively pressing the volar surface of the hand onto the block in 30° of pronation and without pressure. The proximal ulnar stump translates to the volar side and the distance can be measured while pressing. For the contralateral side the transducer was placed dorsally above the DRUJ perpendicular to the longitudinal axis of the ulna. The distance between the dorsal surface of the distal radius and the ulna head were measured (Figure 2). With this ultrasound examination it was possible to measure the dorsal/volar displacement of the ulna head and ulnar stump in addition to the radiological radioulnar convergence.

### Statistical analysis

Mean, median, standard deviation and range were determined for continuous data sets. Group comparisons were performed using the Pearson chi-squared test for categorical variables and the Wilcoxon-rank-sum test, respectively, and the Student's *t*-test for continuous variables. Correlations were carried out with correlation coefficients according to Pearson. *P*-values less than 0.05 and correlation coefficients of 0.50 were considered to be statistically significant.

## Results

Fifteen patients (16 wrists) were available for a follow-up control after an average of 13 years (range 6 to 23). Six patients underwent revision surgery for a painful, unstable ulnar stump; one patient received an ulnar head prosthesis, one a Scheker prosthesis and another four a procedure described by Fernandez. He described a method to stabilize the unstable proximal ulnar stump and proposed the use of an ulnar head prosthesis with its spherical head plunged into a cavity burred into the ulna with the fusion mass intact (Fernandez et al., 2006). Because of failure of the spherical ulna head prothesis, two patients ended up with a one bone forearm, and another one was converted with an ulnar head prosthesis. These six patients were excluded from the clinical, radiographical and sonographic evaluation. Details of the outcomes are summarized in Table 1.

**Table 1.** Complications after SK procedures in 16 wrists (15 patients).

Complications	Wrists	Revision surgery
Hardware irritation	3	Metal removal
Ulnar stump instability	6	Procedure described by Fernandez (*n* = 4), Scheker prosthesis (*n* = 1), ulnar head prosthesis (*n* = 1)
Failure of spherical ulna head prosthesis after Fernandez procedure	3	One bone forearm (*n* = 2), conversion to ulnar head prosthesis (*n* = 1)

### Subjective and clinical results

On the VAS scale, the mean pain was 5 (SD 3.6) with load and 1 (SD 1.6) without load at the follow-up compared with the group of patients after revision surgery with higher pain levels of 8 (SD 2.1) and 3 (SD 2.4), respectively. In addition, the DASH score was 24 (SD 19) and PRWE score 30 (SD 25) after the SK procedure compared with 55 (SD 11) and 64 (SD 21) after revision surgery.

The mean total ROM for pronosupination was 64/64° (SD 25°), which increased to 67/89° (SD 6°) at the follow-up time point. The average grip strength of the operated wrist was 84% compared with the contralateral limb, and force during pronation and supination was 65% and 82%, respectively. Of the 14 employed patients, three were able to resume the same work after the SK procedure.

### Radiological results

No regrowth of the ulnar stump was seen. The arthrodesis between the head of the ulna and the radius had healed and was asymptomatic in all patients. No screw failure was recognized. Three patients had their hardware removed because of pain. Scallop sign was seen in two patients and ectopic ossification in another two. The radioulnar convergence was 8 mm (SD 1.9 mm) without load and 2 mm (SD 1.1 mm) while lifting 1 kg. Four patients could not hold the 2 kg mass during the X-ray, so the data were not analysed further.

There was no significant correlation between the radioulnar convergence and DASH score (*r* = 0.016, *p* = 0.96), PRWE (*r* = −0.1, *p* = 0.78), grip strength (*r* = 0.078, *p* = 0.83) and force during pronation (*r* = 0.38, *p* = 0.28) and supination (*r* = 0.29, *p* = 0.41).

### Sonographic results

The ulnar stump dislocated to the volar side during pressure with a mean of 8 mm (SD 0.7 mm) compared with the contralateral side with a mean of 4 mm (SD 0.7 mm). There was a significant positive strong correlation between sonographically measured ulnar stump displacement and DASH score (*r* = 0.76, *p* = 0.01) and PRWE (*r* = 0.73, *p* = 0.02), respectively. In addition, there was a significant negative correlation between the ulnar stump displacement and grip strength (*r* = −0.64, *p* = 0.05) and force during supination (*r* = −0.71, *p* = 0.02).

In addition, groups with FCU stabilization (*n* = 8), retinaculum flap (*n* = 4) and no stabilization (*n* = 4) were compared, but there was no difference for clinical, sonographic or radiographic parameters (Table S2).

## Discussion

Among 15 patients analysed in this report, six patients required revision surgery and had highly unsatisfactory scores. The other nine patients showed a VAS score of five on load with adequate ROM and grip strength. All patients showed radiographic radioulnar convergence unloaded and while weightbearing. Sonographically, the proximal ulnar stump dislocated volarly by twice the amount compared with the contralateral wrist. A significant correlation between the sonographic ulnar stump instability and clinical scores as the DASH and PRWE as well as supination force and grip strength could be shown, but not for the radiographically measured radioulnar convergence.

Our patients after the SK procedure showed DASH scores of 24, comparable with the results of Giberson-Chen et al. (2020) with DASH scores of 28 after a follow-up of 12 months. Giberson-Chen et al. (2020) reported a postoperative complication incidence of 21%, including revision osteotomy (*n* = 4) and hardware removal (*n* = 4), but no patient required secondary salvage procedures, such as DRUJ arthroplasty. However, the follow-up of 12 months after the SK procedure might be too short to include further revision surgeries. Pronosupination was good in all our patients. Similarly, it does not seem to be a problem in the literature after SK procedures (Carter and Stuart, 2000; Daecke et al., 2004; Fujita et al., 2005; Lluch, 2013). In accordance with the literature, grip strength was satisfactory (Lamey and Fernandez, 1998; Minami et al., 2005).

We measured the proximal ulnar stump instability both radiographically, with the radioulnar convergence, and sonographically. X-ray evaluation showed radioulnar convergence in all remaining patients of 8 mm without weight and 2 mm while lifting 1 kg. Nakamura et al. (1992) and Minami et al. (1995) reported similar results with an average radioulnar convergence distance of 8 mm and 7 mm, respectively. Chu et al. (2008) performed loaded X-rays with a 2.3 kg mass and reported no ulnar stump instability; however, they did not measure the distance between the proximal ulnar stump and the radius.

We found no significant correlation between the radioulnar convergence and clinically measured parameters; but we did find a significant correlation between sonographically measured ulnar stump instability and DASH score, PRWE score, grip strength and force during supination. These results raise the question if the instability of the ulnar stump should not better be determined by sonography rather than by load-bearing radiographs.

Six patients needed revision surgery because of ulnar stump instability in our series. Four patients were treated with a spherical ulnar head prosthesis described by Fernandez et al. (2006). The midterm results of this procedure in 17 patients with an average follow-up of 6 years were encouraging (Fok et al., 2019). In our patient population, three out of four patients had a dislocation of the spherical prosthesis within a few weeks after surgery; two of them ended in a one bone forearm. In the literature, conversion to a one-bone forearm via radioulnar arthrodesis carries a risk of up to 32% nonunion incidence, and results are documented as poorer in younger patients with post-traumatic conditions (Peterson et al., 1995). In our two cases there was no nonunion.

Another salvage technique of the unstable SK procedure was published by Ross et al. (2007). In three patients with unstable ulnar stump, radioulnar pseudarthrosis was revised, ulnar continuity restored with an intercalary graft and forearm rotation restored with matched hemi-resection and interposition arthroplasty at the site of previous radioulnar fusion. All three cases experienced marked functional improvement and resolution of the instability symptoms. We have no experience with this surgical technique.

Verhiel et al. (2019) found comparable long-term patient-reported outcomes of the Darrach and SK procedures with relatively high complication and reoperation incidences (36%). They saw two out of 28 patients (7%) after the SK procedure with proximal ulnar stump instability. However, they reported symptoms of dorsal sensory branch of the ulnar nerve irritability (*n* = 3), heterotopic ossification (*n* = 5), hardware irritation (*n* = 4) and nonunion of pseudarthrosis (*n* = 2) (Table S3).

Lluch (2013) performed the pseudarthrosis at the level of the ulnar head in 70 patients and removed only 5 mm of the bone to reduce the instability of the proximal ulnar stump. Despite resecting only 5 mm, he reported ulnar stump instability in all patients, although it was painless. To prevent ulnar stump instability, some authors have performed a tenodesis with a slip of the ECU or FCU (Breen and Jupiter, 1989; Lamey and Fernandez, 1998).

Three of the six patients who underwent revision surgery had no additional stabilization of the proximal ulnar stump. However, Patient Number 2 had the ulnar stump additionally stabilized with an FCU tendon (50%) but showed on sonography a clear instability of the stump with pronounced pain while weight bearing (VAS 8–10). Conversely, Patient Number 8 had no stabilization and was pain free (DASH 0, PRWE 0, VAS 2). Clinically and radiologically, Kawabata et al. (2010) could not detect any difference in 41 rheumatological patients with and without stabilization of the proximal ulnar stump and concluded that there is no need to stabilize the rheumatoid wrist.

The main limitation of this study is the small number of patients. Another limitation is that loading of the wrist during sonographic evaluation of the ulna stump instability was not standardized. However, the study constitutes a 13-year follow-up report of our series of patients with the SK procedure and reliable outcome measurements, including the objectification of the ulnar stump instability documented by ultrasound and radiograph while weight lifting.

Overall, our study revealed that the SK procedure might not constitute a reliable salvage method for DRUJ disorders. Slater (2008) concluded that complications after the SK procedure are rare. We disagree. Despite stabilization methods, the unstable ulnar stump remains an unsolved problem in long-term follow-up. Because of the high revision incidence after the SK procedure, we suggest performing the SK procedure only in very selected cases.

## References

[bibr1-17531934211004459] Breen TF Jupiter JB . Extensor carpi ulnaris and flexor carpi ulnaris tenodesis of the unstable distal ulna. J Hand Surg Am. 1989, 14: 612–7. 2754191 10.1016/0363-5023(89)90176-7

[bibr2-17531934211004459] Bowers WH . Distal radioulnar joint arthroplasty: the hemiresection-interposition technique. J Hand Surg Am. 1985, 10: 169–78. 3980927 10.1016/s0363-5023(85)80100-3

[bibr3-17531934211004459] Carter PB Stuart PR . The Sauve-Kapandji procedure for posttraumatic disorders of the distal radioulnar joint. J Bone Joint Surg Br. 2000, 82: 1013–8. 11041592 10.1302/0301-620x.82b7.10674

[bibr4-17531934211004459] Chu PJ Lee HM Hung ST Shih JT . Stabilization of the proximal ulnar stump after the Darrach or Sauvé-Kapandji procedure by using the extensor carpi ulnaris tendon. Hand. 2008, 3: 346–51. 18780014 10.1007/s11552-008-9113-3 PMC2584221

[bibr5-17531934211004459] Daecke W Streich NA Martini AK . The Sauvé-Kapandji operation. Indications and results. Orthopade. 2004, 107: 1057–64. 10.1007/s00132-004-0657-9 15127198

[bibr6-17531934211004459] Darrach W . Partial excision of the lower shaft of the ulna for deformity following Colles’ fracture. Ann Surg. 1913, 57: 764–5. 1735229

[bibr7-17531934211004459] Del Pino JG Fernandez DL . Salvage procedure failed Bowers’ hemiresection interposition technique in the distal radioulnar joint. J Hand Surg Br. 1998, 23: 749–53. 9888674 10.1016/s0266-7681(98)80089-2

[bibr8-17531934211004459] Fernandez DL Soneschild ES Abella DM . Treatment of failed Sauvé-Kapandji procedures with a spherical ulnar head prosthesis. Clin Orthop Relat Res. 2006, 445: 100–7. 16601411 10.1097/01.blo.0000205901.13609.70

[bibr9-17531934211004459] Fok MWM Fernandez DL van Schoonhoven J . Midterm outcomes of the use of a spherical ulnar head prosthesis for failed Sauvé-Kapandji procedures. J Hand Surg Am. 2019, 44: 66.e1–9. 29934080 10.1016/j.jhsa.2018.05.005

[bibr10-17531934211004459] Fujita S Masada K Takeuchi E Yasuda M Komatsubara Y Hashimoto H . Modified Sauvé-Kapandji procedure for disorders of the distal radioulnar joint in patients with rheumatoid arthritis. J Bone Joint Surg Am. 2005, 87: 134–9. 15634824 10.2106/JBJS.C.01600

[bibr12-17531934211004459] Giberson-Chen CC Leland HA Benavent KA Herper CM Earp BE Rozental TD . Functional outcomes after Sauvé-Kapandji arthrodesis. J Hand Surg Am. 2020, 45: 408–16. 31948706 10.1016/j.jhsa.2019.11.014

[bibr13-17531934211004459] Herbert TJ van Schoonhoven J . Ulnar head replacement. Tech Hand Up Extrem Surg. 2007, 11: 98–108. 17536532 10.1097/bth.0b013e318033738a

[bibr14-17531934211004459] Hess F Farshad M Sutter R Nagy L Schweizer A . A novel technique for detecting instability of the distal radioulnar joint in complete triangular fibrocartilage complex lesions. J Wrist Surg. 2012, 1: 153–8. 24179720 10.1055/s-0032-1312046 PMC3658687

[bibr15-17531934211004459] Hudak PL Amadio PC Bombardier C . Development of an upper extremity outcome measure: the DASH (Disabilities of the Arm, Shoulder and Hand). Am J Ind Med. 1996, 29: 602–8. 8773720 10.1002/(SICI)1097-0274(199606)29:6<602::AID-AJIM4>3.0.CO;2-L

[bibr16-17531934211004459] Kawabata A Egli T Hashimoto H Hasada K Saito S . A comparative study of the modified Sauvé-Kapandji procedure for rheumatoid wrist with and without stabilization of the proximal ulnar stump. J Hand Surg Eur. 2010, 35: 659–63. 10.1177/1753193410367599 20351133

[bibr18-17531934211004459] Lamey DM Fernandez DL . Results of the modified Sauvé-Kapandji procedure in the treatment of chronic posttraumatic derangement of the distal radioulnar joint. J Bone Joint Surg Am. 1998, 80: 1758–69. 9875933 10.2106/00004623-199812000-00005

[bibr19-17531934211004459] Lees VC Scheker LR . The radiological demonstration of dynamic ulnar impingement. J Hand Surg Br. 1997, 22: 448–50.

[bibr20-17531934211004459] Lluch A . The Sauvé-Kapandji procedure. J Wrist Surg. 2013, 2: 33–40. 24436787 10.1055/s-0032-1333465 PMC3656576

[bibr21-17531934211004459] MacDermid JC Turgeon T Richards RS Beadle M Roth JH . Patient rating of wrist pain and disability: a reliable and valid measurement tool. J Orthop Trauma. 1998, 12: 577–86. 9840793 10.1097/00005131-199811000-00009

[bibr22-17531934211004459] Masaoka S Longsworth SH Werner FW Short WH Green JK . Biomechanical analysis of two ulnar head prostheses. J Hand Surg Am. 2002, 27: 845–53. 12239675 10.1053/jhsu.2002.34010

[bibr23-17531934211004459] Minami A Iwasaki N Ishikawa J Suenaga N Kato H . Stabilization of the proximal ulnar stump in the Sauvé-Kapandji procedure by using the extensor carpi ulnar tendon: long-term follow-up studies. J Hand Surg Am. 2006, 31: 440–4. 16516739 10.1016/j.jhsa.2005.11.012

[bibr24-17531934211004459] Minami A Iwasaki N Ishikawa J Suenaga N Yasudo K Kato H . Treatments of osteoarthritis of the distal radioulnar joint: long-term results of three procedures. J Hand Surg Am. 2005, 10: 243–8. 10.1142/S0218810405002942 16568521

[bibr25-17531934211004459] Minami A Kato H Iwasaki N . Modification of the Sauvé-Kapandji procedure with extensor carpi ulnaris tenodesis. J Hand Surg Am. 2000, 25: 1080–4. 11119666 10.1053/jhsu.2000.20158

[bibr26-17531934211004459] Minami A Suzuki K Suenaga N Ishikawa J . The Sauvé-Kapandji procedure for osteoarthritis of the distal radioulnar joint. J Hand Surg Am. 1995, 20: 602–8. 7594287 10.1016/s0363-5023(05)80276-x

[bibr27-17531934211004459] Nakamura R Tsunoda K Watanabe K Horii E Miura T . The Sauvé-Kapandji procedure for chronic dislocation of the distal radio-ulnar joint with destruction of the articular surface. J Hand Surg Br. 1992, 17: 127–32. 1588189 10.1016/0266-7681(92)90071-9

[bibr28-17531934211004459] Peterson C Maki S Wood M . Clinical results of the one-bone-forearm. J Hand Surg Am. 1995, 20: 609–18. 7594288 10.1016/S0363-5023(05)80277-1

[bibr29-17531934211004459] Reissner L Böttger K Klein HJ Calcagni M Giesen T . Midterm results of semiconstrained distal radioulnar joint arthroplasty and analysis of complications. J Wrist Surg. 2016, 5: 290–6. 27777820 10.1055/s-0036-1583303 PMC5074829

[bibr30-17531934211004459] Ross M Thomas J Couzens G Coleman S . Salvage of unstable Sauvé-Kapandji procedure: a new technique. Tech Hand Up Extrem Surg. 2007, 11: 87–92. 17536530 10.1097/bth.0b013e318033b537

[bibr31-17531934211004459] Sauvé L Kapandji M . Nouvelle technique de traitement chirurgical des luxation récidivantes isolées de l’extrémité inférieure de cubitus. J Chir (Paris). 1936, 47: 589–94.

[bibr32-17531934211004459] Scheker LR . Implant arthroplasty for the distal radioulnar joint. J Hand Surg Am. 2008, 33: 1639–44. 18984351 10.1016/j.jhsa.2008.08.014

[bibr33-17531934211004459] Scheker LR Babb BA Killion PE . Distal ulnar prosthetic replacement. Orthop Clin North Am. 2001, 32: 365–76. 11331548 10.1016/s0030-5898(05)70256-x

[bibr34-17531934211004459] Slater RR . The Sauvé-Kapandji procedure. J Hand Surg Am. 2008, 33: 1632–8. 18984350 10.1016/j.jhsa.2008.08.011

[bibr35-17531934211004459] Sotereanos DG Papatheodorou LK Williams BG . Tendon allograft interposition for failed distal ulnar resection: 2- to 14-year follow-up. J Hand Surg Am. 2014, 39: 443–8.e1. 24359796 10.1016/j.jhsa.2013.11.004

[bibr36-17531934211004459] Tang JB . Re: Levels of experience of surgeons in clinical studies. J Hand Surg Eur. 2009, 34: 137–8. 10.1177/17531934097321 19270008

[bibr37-17531934211004459] Tang JB Giddins G . Why and how to report surgeons’ levels of expertise. J Hand Surg Eur. 2016, 41: 365–6. 10.1177/1753193416641590 27076602

[bibr38-17531934211004459] Verhiell SHWL Özkan S Ritt MJPF Chen NC Eberlin KR . A comparative study between Darrach and Sauvé-Kapandji procedures for post-traumatic distal radioulnar joint dysfuncion. Hand (NY). 2019, 27: 1558944719855447. DOI: 10.1177/1558944719855447. 10.1177/1558944719855447 PMC8120580 31248288

[bibr39-17531934211004459] Watson HK Ryu J Burgess RC . Matched distal ulnar resection. J Hand Surg Am. 1986, 11: 812–7. 3794235 10.1016/s0363-5023(86)80228-3

